# IFN Signaling: How a Non-Canonical Model Led to the Development of IFN Mimetics

**DOI:** 10.3389/fimmu.2013.00202

**Published:** 2013-07-25

**Authors:** Howard M. Johnson, Ezra Neptune Noon-Song, Rea Dabelic, Chulbul M. Ahmed

**Affiliations:** ^1^Department of Microbiology and Cell Science, University of Florida, Gainesville, FL, USA; ^2^Department of Microbiology and Immunology, Columbia University, New York, NY, USA

**Keywords:** cytokines, JAK/STAT signaling, interferon mimetics, interferon receptor, interferon signaling

## Abstract

The classical model of cytokine signaling dominates our view of specific gene activation by cytokines such as the interferons (IFNs). The importance of the model extends beyond cytokines and applies to hormones such as growth hormone (GH) and insulin, and growth factors such as epidermal growth factor (EGF) and fibroblast growth factor (FGF). According to this model, ligand activates the cell via interaction with the extracellular domain of the receptor. This results in activation of receptor or receptor-associated tyrosine kinases, primarily of the Janus activated kinase (JAK) family, phosphorylation and dimerization of the signal transducer and activator of transcription (STAT) transcription factors, which dissociate from the receptor cytoplasmic domain and translocate to the nucleus. This view ascribes no further role to the ligand, JAK kinase, or receptor in either specific gene activation or the associated epigenetic events. The presence of dimeric STATs in the nucleus essentially explains it all. Our studies have resulted in the development of a non-canonical, more complex model of IFNγ signaling that is akin to that of steroid hormone (SH)/steroid receptor (SR) signaling. We have shown that ligand, receptor, activated JAKs, and STATs are associated with specific gene activation, where the receptor subunit IFNGR1 functions as a co-transcription factor and the JAKs are involved in associated epigenetic events. We found that the type I IFN system functions similarly. The fact that GH receptor, insulin receptor, EGF receptor, and FGF receptor undergo nuclear translocation upon ligand binding suggests that they may also function similarly. The SH/SR nature of type I and II IFN signaling provides insight into the specificity of signaling by members of cytokine families. The non-canonical model could also provide better understanding to more complex cytokine families such as those of IL-2 and IL-12, whose members often use the same JAKs and STATs, but also have different functions and properties.

## Introduction

The classical Janus activated kinase (JAK)/signal transducer and activator of transcription (STAT) model of cytokine signaling dominates our view of specific gene activation by cytokines such as the interferons (IFNs) (1). The importance of the model extends to complex cytokine families such as those of IL-2 (2) and IL-12 (3), as well as to hormones such as prolactin and angiotensin and growth factors such as growth hormone (GH) and platelet-derived growth factor (4–6). In this model, ligand activates the cell solely via interaction with the extracellular domain of the receptor complex (1, 7). This in turn results in the activation of receptor or receptor-associated tyrosine kinases, primarily of the JAK family, leading to phosphorylation and dimerization of the STAT transcription factors, which then dissociate from the receptor cytoplasmic domain and translocate to the nucleus. This view ascribes no further role to the ligand, JAK kinase, or the receptor in the signaling process.

It has recently been acknowledged that the classical model of JAK/STAT signaling was over-simplified in its original form. In the case of IFNγ, complexity beyond simple JAK/STAT activation is indicated in the relatively recent demonstration that other pathways, including mitogen activated protein kinase (MAPK), phosphoinositide 3-kinase (PI3K), Ca^2+^/calmodulin (CaM) kinase II, nuclear factor-κB (NF-κB), and others cooperate with or act in parallel to that of JAK/STAT signaling to regulate IFNγ effects at the level of gene activation and cell phenotypes (7). All of the pathways are generic in the sense that a plethora of cytokines, hormones, and growth factors with functions different from those of IFNγ also activate them.

There is evidence that JAK kinases, including the mutant JAK2V617F, play an important role in the epigenetics of gene activation in addition to STAT activation in the cytoplasm (8). Leukemic cells with a JAK2V617F gain-of-function mutation have constitutively active JAK2V617F in the nucleus. This leads to phosphorylation of tyrosine 41 (Y41) on histone H3, which results in dissociation of heterochromatin protein 1α (HP1α). The resultant heterochromatin remodeling was associated with exposure of euchromatin for gene activation. Although present in the nucleus, wild-type JAK2 was only activated when K562 cells were treated with PDGF or LIF, or when BaF3 cells were treated with IL-3. The question of how a ligand/receptor interaction resulted in the presence of activated JAK2, pJAK2, in the nucleus was not addressed, nor its targeting mechanism to discrete genomic sites and specific promoters.

Signal transducer and activator of transcriptions form dimers when activated, but there are only seven different types of STATs, and the dimers are predominantly homomeric in nature. Given that there are functionally over 60 different types of ligands that use STATs, it is difficult to decipher the mechanism of their different specificities solely in the context of the particular STATs involved (9–11). Further, some ligands such as IFNγ and IL-10 use STAT1α, while other factors such as GH, IL-2, and IL-7 use STAT5β, but these molecules have different effects on cells (9, 12). The classical model in effect gives the STAT some sort of Lamarchian power where it “knows” the ligand responsible for its activation and proceeds on its own to activate specific genes as well as directing specific epigenetic events associated with the gene activation. Thus, the classical JAK/STAT model tells us very little about the unique aspects of IFNγ signaling or the basis of type I IFN signaling where up to 20 or more IFNs all interact with the same heterodimeric receptor complex and activate the same STAT transcription factors, but vary in functions such as antiproliferative activity and apoptosis (10, 11, 13). Translationally, the lack of understanding of mechanism makes it very difficult to deal with the mixed effects of type I IFNs as therapeutics (14).

Focusing initially on IFNγ, we have shown that ligand, receptor, and activated JAKs are involved in nuclear events that are associated with specific gene activation, where the receptor subunit IFNGR1 functioned as a transcription/co-transcription factor and the JAKs exerted key epigenetic phosphorylation of histone H3 at tyrosine 41 (H3pY41) (15–17). We showed that the N-terminus of IFNγ played an important role in extracellular recognition of receptor, but unexpectedly in the process of endocytosis, the C-terminus of IFNγ interacted with a specific site in the cytoplasmic domain of receptor in the IFNGR1 subunit (18). This led to development of a mimetic involving the C-terminus of IFNγ (18). The complex of IFNγ or mimetic with IFNGR1, activated STAT1α, and activated JAKs 1 and 2 underwent nuclear translocation for specific gene activation (16). Details of critical aspects of these studies are provided below. Type I IFNs play a key role in innate and adaptive immunity with a role in both antiviral defense and autoimmunity. We have recently shown similarities and differences between type I and type II IFN signaling in terms of IFN/receptor/STAT/JAK complexes in the nucleus and intracellular receptor interaction.

Peptide mimetics have been increasingly manufactured by the pharmaceutical industry due to their versatility and high biological activity. They offer advantages such as high specificity for their targets, and low toxicity. Peptides can vary in length from three to over 60 amino acids, and thus have a broad range of structural properties. There are already several peptide mimetics on the market to treat a wide range of diseases, such as boceprevir to treat hepatitis C, romidepsin as an anti-cancer drug, and liraglutide to treat Type 2 diabetes (19). The non-canonical model of IFNγ signaling presented here has been key to the development of IFN mimetics.

## Classical Model of JAK/STAT Signaling in the Context of IFNγ

**Figure 1:**
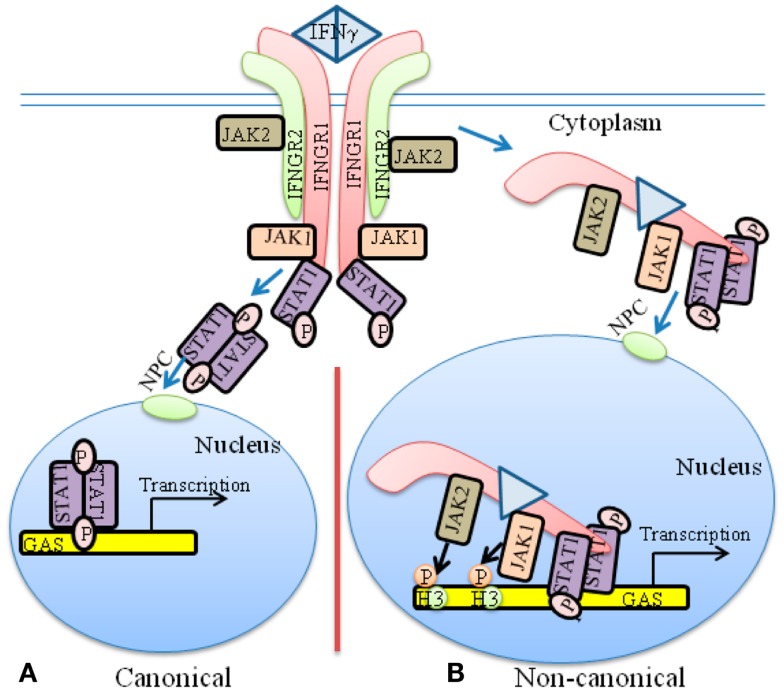
**The classical and non-canonical models of IFNγ signaling**. **(A)** In the classical model of IFNγ signaling, dimeric IFNγ cross-links the IFNGR1 receptor subunit that results in allosteric changes in receptor cytoplasmic domain. This results in movement of JAK2 from receptor subunit IFNGR2 to IFNGR1. The JAKs autophosphorylate and then phosphorylate IFNGR1 cytoplasmic domain. This results in binding, phosphorylation, and dimer formation of STAT1α. The dimeric STAT1α dissociates from receptor and undergoes nuclear translocation via an intrinsic NLS for specific gene activation. **(B)** The non-canonical model of IFNγ signaling involves IFNγ binding to receptor extracellular domain, followed by movement to IFNGR1 cytoplasmic domain in conjunction with endocytosis. The cytoplasmic binding increases the affinity of JAK2 for IFNGR1, which is the basis for its movement to IFNGR1. This results in autoactivation of the JAKs, phosphorylation of IFNGR1 cytoplasmic domain, and the binding and phosphorylation of STAT1α at IFNGR1. The complex of IFNGR1/STAT1α/JAK1/JAK2 undergoes active nuclear transport where the classic polycationic NLS of IFNγ plays a key role for this transport to genes in the nucleus that are specifically activated by IFNγ. Details of the non-canonical model are presented in the text. GAS, IFN gamma activated sequence; H3, histone H3; NPC, Nuclear pore complex.

The classical model of JAK/STAT signaling for IFNγ is illustrative of the weight that it puts upon the STATs in specific gene activation (Figure 1A). The heterodimeric receptor subunits are IFNGR1 and IFNGR2, respectively (7, 20, 21). An asymmetric dimer of IFNγ binds predominantly to and cross-links the extracellular domains of two IFNGR1 chains. The model contends that the cross-linking initiates allosteric changes in receptor cytoplasmic domains that are responsible for subsequent events. JAK1 is associated with IFNGR1, whereas JAK2 is associated with IFNGR2. The extracellular binding of IFNγ to IFNGR1 is somehow responsible for the movement of JAK2 from IFNGR2 to IFNGR1, where a sequence of events causes autophosphorylation of the JAKs and tyrosine phosphorylation of IFNGR1, followed by recruitment and phosphorylation of STAT1α (pSTAT1α) at IFNGR1. According to the model, pSTAT1α forms a dimer, dissociates from IFNGR1 and goes to the nucleus via an intrinsic nuclear localization sequence (NLS).

Structure studies have shown that dimeric pSTAT1α binds to the GAS element of the IFNγ promoter (22), which has been interpreted as validation of the classical model. There are concerns, however, with this leap in logic. It has been shown, for example, that contrary to the requirement of cross-linking of receptor by IFNγ dimer, monomeric IFNγ can also stimulate the activation of STAT1α (23, 24). Additionally, the classical JAK/STAT model does not explain the basis or mechanism for movement of JAK2 from IFNGR2 to IFNGR1 beyond the interaction of IFNγ with IFNGR1 extracellular domain. Similarly, the recent observations that activated JAKs undergo nuclear translocation and possess epigenetic function are not dealt with by the classical model in the context of the activating ligand (8). The fact that these activated nuclear JAKs are involved in important epigenetic events is thus disconnected from the classical model of JAK/STAT signaling by cytokines such as the IFNs (1, 21). The issues raised above concerning the classical JAK/STAT model along with the suggestion by the model that the activated STATs possess the intrinsic property of determining the specificity of cytokine and other factor signaling is of particular concern and is dealt with in some detail in the non-canonical signaling model that we have developed, including the development of IFN mimetics. Rational development of IFN mimetics based on the classical model, for example, has not been reported based on cross-linking the receptor extracellular domains. Thus, it is revealing that little insight concerning such issues is contained within the classical model of JAK/STAT signaling.

## Activated JAKs in the Nucleus: On Their Own or Team Players?

At the STAT level, there is recent evidence of a functional interaction between different STATs in gene activation/suppression. Induction of STAT5 phosphorylation by interleukin 2 (IL-2) resulted in more binding of STAT5 and less binding of STAT3 at similar DNA sites, whereas phosphorylation of STAT3 by IL-6 induced the opposite; the combination of the two STATs resulted in dynamic regulation of the IL-17 gene locus by the opposing effects of IL-2 (STAT5) and IL-6 (STAT3) (25, 26). These Yin–Yang interactions of STAT transcription factors are referred to as “specification” with respect to lymphocyte phenotypes. How these STAT interactions at the level of DNA binding translate into specific gene activation by the inducing cytokine was not obvious. The recent report of activated JAK2 in the nucleus performing an epigenetic function is potentially very important for the following reasons. First, the activated JAK2 was shown to perform the epigenetic function of phosphorylation of tyrosine 41 on histone H3 (8). Second, it is highly unlikely that the activated JAK2 is acting randomly in the nucleus, so how and with what are its epigenetic functions coordinated?

We will first address aspects of what activated JAKs do in the nucleus. The epigenetic finding indicated above focused primarily on leukemic cells where a mutated JAK2, JAK2V617F, with a gain-of-function is found in the nucleus (8). Constitutively active JAK2V617F was shown to phosphorylate histone H3 on tyrosine 41 (H3pY41), which led to dissociation of HP1α from H3. The resultant heterochromatin remodeling was associated with exposure of euchromatin for gene activation. Wild-type JAK2 was shown to be constitutively present in the nucleus of cells also, but unlike JAK2V617F, was only activated when K562 cells were treated with the growth factors platelet-derived growth factor or leukemia inhibitory factor, or when BaF3 cells were treated with the cytokine IL-3. A key question is whether the nuclear H3 phosphorylations are random or under control by factors associated with the activating cytokine?

This question was addressed in IFN studies by treating cells with IFNγ and tracking activated JAK2 in the nucleus (16). Using chromatin immunoprecipitation (ChIP) analysis, it was shown that activated JAK2 (pJAK2) and H3pY41 were associated with the GAS promoter element at the IRF-1 gene, a gene that is activated by IFNγ (16). pJAK1 was also associated with the IRF-1 GAS element. None of these factors were associated with the promoter of the β-actin gene, a gene not affected by IFNγ. A similar result was observed with TYK2 in IFNα treated cells where TYK2 and H3pY41 were present at the promoter of the oligoadenylate synthetase (OAS) 1 gene, a gene activated by type I IFNs, but not at the promoter of the β-actin gene (17). It is important to note that ChIP analysis also showed the presence of STAT1 at the IRF1 and OAS1 promoters of IFNγ and/or IFNα treated cells, but not at the β-actin promoter. This would suggest that the activated JAKs and the STATs track to the same promoters, which would suggest that their nuclear activities are coordinated.

The above result with JAK2V617F, pJAK2, and H3pY41 is not the first report of JAKs in the nucleus. For example, JAK1, JAK2, and TYK2 have all been previously reported to be constitutively present in the nucleus (27–32). Also, GH has been shown to induce the translocation of pJAK2 to the nucleus in GH receptor transfected CHO (27) and CWSV-1 (33) cells. None of these studies, however, provided the functional significance to an activated JAK in the nucleus such as that contained in the JAK2 H3pY41 finding (8).

The fact that the hematological disorders associated with JAK2V617F show characteristic phenotypic similarities would suggest that the epigenetic activity of JAK2V617F occurs in association with the relevant hematological receptor. It is of interest therefore that it has been shown that JAK2V617F activation required the association of the mutant JAK2 with a homodimeric type I cytokine receptor (34, 35). Specifically, erythropoietin receptor, thrombopoietin receptor, or granulocyte colony-stimulating receptor are required for hormone/growth factor independent activation of JAK2V617F. This raises the question of whether there are receptor/JAK2V617F complexes in the vicinity of promoters of genes that are activated in cancers caused by or associated with JAK2V617F? All of this has implications for how particular tyrosine kinases cause or are associated with specific cancers.

Studies in a hematopoietic tumor model in *Drosophila* with a hyperactive JAK kinase mutant (Tum-1) of the wild-type JAK, Hopscotch (Hop), showed a high incidence of hematopoietic tumors (36). Tum-1 caused hyperphosphorylation of the *Drosophila* STAT, STAT92E, which was associated with tumor induction. Reduction in the dose of STAT92E gene had a suppressive effect on Tum-1 tumorigenicity. It was also shown that the JAK (Tum-1) overactivity globally disrupted heterochromatin gene silencing and that overexpression of *Drosophila* HP1 suppressed JAK-induced tumors. Conversely, mutations in the HPI gene enhanced the Tum-1 oncogenic JAK kinase. These results are consistent with and were further clarified by the heterochromatin destabilization that was caused by the H3Y41-to-H3pY41 effects of JAK2V617F that resulted in dissociation of HP1α from H3 (8).

## A Non-Canonical Model of Type I and Type II IFN Signaling

Both IFNγ and type I IFNs such as IFNα have been shown to function intracellularly, activate their respective STATs, and to translocate to the nucleus of receptor-expressing cells (37, 38). It was previously shown that IFNγ and one of its receptor subunits, IFNGR1, are translocated to the nucleus together with activated STAT1α (17, 39). Active nuclear transport depended on a polycationic NLS in the C-terminus of IFNγ, the nuclear import proteins importins α and β, and ATP/GTP as an energy source (40, 41).

The nuclear targets of IFNγ and IFNGR1 were also identified (15, 16). By ChIP followed by PCR, IFNγ, its receptor subunit IFNGR1, and STAT1α were found to be associated with the IFNγ-activated sequence (GAS) element in the promoter of two genes stimulated by IFNγ. Examination of nuclear extracts from IFNγ treated WISH cells showed that IFNγ, IFNGR1, and STAT1α proteins were associated with the GAS promoter. The same associations were also demonstrated by electrophoretic mobility shift assay (EMSA). Transfection with a GAS-luciferase gene together with the IFNGR1 and non-secreted IFNγ resulted in enhanced promoter activity. Additionally, IFNGR1 fused to the yeast GAL-4 DNA binding domain resulted in enhanced transcription from the GAL-4 response element in IFNγ treated cells, suggesting the presence of a transactivation domain in IFNGR1. These nuclear studies suggest a transcriptional/co-transcriptional role for IFNGR1, which may provide insight into the specificity of IFNγ signaling. A model for these non-canonical IFNγ signaling events is presented in Figure 1B.

**Figure 2:**
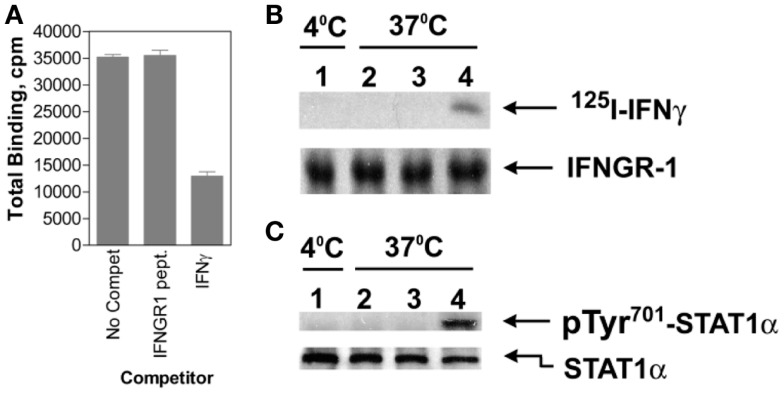
**IFNγ moves from IFNGR1 extracellular domain to its cytoplasmic domain during endocytosis**. **(A)** Binding of ^125^I-IFNγ (10 nM) to IFNGR on P_1_388D cells in the presence of unlabeled IFNγ (30 nM) or IFNGR1(253–287) (1 μM) at 4°C for 30 min. The IFNGR1 (253–287) peptide corresponds to IFNGR1 intracellular binding site for IFNγ. IFNγ but not IFNGR1 (253–287) blocked extracellular ^125^I-IFNγ binding. **(B)** In lane 1, ^125^I-IFNγ was incubated along with P_1_388D cells at 4°C for 5 min. In lanes 2 and 3, cells were first incubated with 25 and 50 μM, respectively of IFNGR1(253–287) for 5 min at 37°C to facilitate internalization. Cells were then washed and incubated alone with ^125^I-IFNγ (5 nM) at 37°C for 5 min. In lane 4, cells were incubated alone with ^125^I-IFNγ at 37°C for 5 min. All of the cells were then acid washed at 4°C to remove surface ^125^I-IFNγ, lysed, and the extracts were immunoprecipitated with antibodies to IFNGR1. Precipitated IFNGR1 was transferred to nitrocellulose membranes and electrophoresed. The membranes were subjected to autoradiography and blotted for IFNGR1 protein. Note that intracellular IFNGR1(253–287) blocked ^125^I-IFNγ binding to IFNGR1 intracellular domain. **(C)** Same as for **(B)**, except extracts were immunoprecipitated with antibodies to STAT1α and blotted for pTyr^701^- STAT1α and STAT1α. See Ahmed et al. (41) for details.

Cytokines such as IFNs are assumed to bind solely to the receptor extracellular domain, resulting in allosteric changes on the cytoplasmic domain that initiates signaling events. It was shown, however, that IFNγ bound first to IFNGR1 extracellular domain involving in part its N-terminus and then, during endocytosis, to IFNGR1 cytoplasmic domain via its C-terminus as described below and in Figure 2 (41). This was shown as follows. An intracellular excess of a peptide representing the cytoplasmic binding site on IFNGR1 for the C-terminus of IFNγ, IFNGR1 (253–287), prevented the complexation of internalized IFNγ with the cytoplasmic domain of cell-surface IFNGR1 in cells that were actively internalizing IFNγ (41). Moreover, such cells were also blocked with respect to the tyrosine phosphorylation of STAT1α. Thus, internalized IFNγ appeared to be able to interact with the cytoplasmic domain of IFNGR1 in intact cells as part of the signal transduction events leading to STAT1α tyrosine phosphorylation. Since the IFNGR1 cytoplasmic domain would be present on the outer surface of the endocytic vesicle following endocytosis, this would suggest that IFNγ can traverse the membrane of the endocytic vesicle during internalization to contact the cytoplasmic domain of IFNGR1. Cytosolic injection of antibodies to IFNγ C-terminal amino acids 95–132 blocked STAT1α nuclear translocation in response to extracellular IFNγ (40), consistent with these observations. This further supports the idea that the C-terminus of endocytosed IFNγ accesses the cytosol, although the mechanism is as yet undetermined.

As indicated earlier, JAK2 moves from the cytoplasmic domain of IFNGR2 to IFNGR1 in cells treated with IFNγ via a previously unknown mechanism. Binding of IFNγ to the 253–287 region of IFNGR1 was shown to enhance the binding of JAK2 to an adjacent site on IFNGR1 cytoplasmic domain (42). Thus, the movement of JAK2 from IFNGR2 to IFNGR1 cytoplasmic domain seems to be explainable by the exercise of the law of mass action based on the increased binding affinity of IFNGR1 cytoplasmic domain for JAK2 (42).

All of the above IFNγ effects can be replicated by peptides corresponding to the internalized C-terminus residues 95–132 of mouse IFNγ or residues 95–134 of human IFNγ (43). Thus, peptides corresponding to these residues, mIFNγ(95–132) and hIFNγ(95–134) respectively, with a palmitate attached for cell penetration, function as IFNγ mimetics. The properties and uses of these IFNγ mimetics are described in detail below. It is noteworthy that there are no IFN or other cytokine mimetics based on extracellular recognition and cross-linking of receptor chains as per the classical model.

Recently, insight has been gleaned on the intracellular aspects of type I IFN signaling. It was shown by western blotting of nuclear extracts that type I IFN signaling involves activated TYK2 in the nucleus, similar to pJAK2 in the nucleus of IFNγ treated cells (17). The nucleus of WISH cells contained constitutively expressed non-phosphorylated TYK2, but activated TYK2, pTYK2, as well as pJAK1 was found in the nucleus of cells only after treatment with type I IFNs IFNα or IFNτ. Both activated STAT1 and STAT2 were present in the nucleus of cells treated with type I IFNs. With IFNγ, only the receptor subunit IFNGR1 underwent nuclear translocation in IFNγ treated cells, but both receptor subunits IFNAR1 and IFNAR2 underwent nuclear translocation in type I IFN treated cells as determined by western blotting of nuclear extracts and confocal microscopy of GFP-receptor fusion proteins. The GFP-IFNτ fusion protein also underwent nuclear import, thus demonstrating that type I IFNs also translocated to the nucleus.

With all of these components of the type I IFN signaling system in the nucleus, there was interest in determining where they went in terms of promoters and whether they were associated with each other for some coordinate nuclear function. Therefore, ChIP-qPCR assays were performed to determine if the type I IFN players were specifically recruited to the promoter region of a gene activated by IFNα in cells (17). The promoter region of the OAS1 gene, which has an IFN sensitive response element (ISRE) and is involved in IFN antiviral activity, was thus examined (17). IFNAR1, IFNAR2, TYK2, pSTAT1, and H3pY41 were found at the OAS1 promoter, but not at the β-actin promoter, a gene that is not directly affected by type I IFNs. Consistent with the ChIP data, immunoprecipitation of IFNAR1 in nuclear extracts of IFNα treated cells, followed by Western blotting showed TYK2, pSTAT1, and H3pY41 associated with IFNAR1. Thus, the various players in type I IFN signaling were found associated in the nucleus of IFN treated cells specifically at the promoter of a key gene in IFN antiviral activity.

Given the specific epigenetic events that are associated with gene activation, ChIP analysis was used to monitor demethylation/acetylation of lysine 9 on histone H3 (17). Type I IFN treated cells showed decreased trimethylated lysine on H3, H3K9me3, in the OAS1 promoter region of cells. Acetylation of H3K9, H3K9ac, occurred concomitantly over the same time span. Demethylation/acetylation of H3K9 is associated with gene activation (44, 45). Related to this, phosphorylation of H3 at Y41, H3pY41, increased as H3K9me3 decreased over the same time period. By comparison, the constitutively activated β-actin gene, which is not affected by IFN, showed constitutive H3K9ac, no H3pY41, and no H3K9me3. The nuclear trafficking and activities at specific genes that are associated with treatment of cells with IFN suggest that the receptor/transcription factor/JAK complex plays a key role in specific gene activation, including the related heterochromatin modifications.

## From Non-Canonical Signaling to IFN Mimetic Development

Attachment of the fatty acid palmitic acid (lipo-) to the IFNγ peptides for cell penetration conveyed IFN signaling properties (46). Lipo-mIFNγ(95–132) and lipo-hIFNγ(95–134) possess classical polycationic NLSs in their C-terminus and an alpha helix in their N-terminus that were required for their mimetic activity (46, 47). The mimetics activated STAT1α and induced its translocation to the nucleus (48), and also possessed transactivational activity at the GAS promoter, demonstrating that they functioned similar to IFNγ in the nucleus (15). The mimetics possessed potent antiviral activity against vesicular stomatitis virus (VSV) and encephalomyocarditis virus (EMCV), and like IFNγ they induced increased expression of MHC class II antigens on macrophages (18). The induction of antiviral activity was confirmed by others (49).

A stringent test of the mimetic in terms of antiviral activity was observed with a poxvirus, vaccinia virus, which is used worldwide to vaccinate against smallpox infections, and is a prototype of the poxvirus family (50). These viruses are particularly effective in neutralizing host innate antiviral defense mechanisms, such as the IFN system, because they produce soluble secreted proteins that bind to and prevent IFNα, IFNβ, and IFNγ from binding to their respective receptors on the cell membrane (50). An important virulence factor of vaccinia virus is the B8R protein, which is a homolog of the extracellular domain of the IFNγ receptor and can therefore bind to intact IFNγ and prevent its interaction with the receptor (51). It was hypothesized that the IFNγ mimetics would bypass the poxvirus virulence factor B8R protein that binds to intact IFNγ, thus preventing its interaction with the receptor. Human and murine IFNγ mimetic peptides were introduced into an adenoviral vector for intracellular expression. Murine IFNγ mimetic peptide, lipo-mIFNγ(95–132), was also expressed via chemical synthesis with attached palmitic acid for penetration of cell plasma membrane. In contrast to the intact human IFNγ, the mimetics did not bind poxvirus B8R protein. Expression of B8R protein in epithelial WISH cells did not block the antiviral effect of the mimetics against EMCV or VSV, while the antiviral activity of human IFNγ was neutralized. Consistent with the antiviral activity, the upregulation of MHC class I molecules on WISH cells by the IFNγ mimetics was not affected by B8R protein, while IFNγ induced upregulation was blocked. Finally, the mimetics, but not IFNγ, inhibited vaccinia virus replication in African green monkey kidney BSC-40 cells. The small peptide mimetics of IFNγ can avoid the B8R virulence factor for poxviruses and thus are potential candidates for antivirals against smallpox virus (43, 46, 48).

It was further shown that lipo-mIFNγ(95–132) protected C57BL/6 mice against overwhelming lethal vaccinia virus infection (48). Control mice died at 9–10 days post infection, but intraperitoneal injection of the mimetic as late as 6 days post infection resulted in 40 percent protection. Administration of mimetic by the oral route also completely protected mice against the intranasal route of a lethal dose of vaccinia virus challenge. In addition to the direct antiviral effects, the mimetic also possessed adjuvant effects in boosting humoral and cellular immunity. This combination of antiviral and adjuvant effects by the IFN mimetic probably played a role in its potent anti-vaccinia virus properties. IFNγ is generally not extensively used as a therapeutic, the reason for which is not well understood. It should be noted that the presence of receptors on a large number of cells could serve as a “sink,” thus affecting access of IFNγ to sites and cells for which it was intended. The IFNγ mimetics do not recognize the receptor extracellular domain and thus could possibly have better access to intended targets.

The pattern of nuclear signaling by type I IFNs is similar to that of IFNγ nuclear signaling (11). Thus, in order to determine if IFNα1 and IFNβ possessed similar C-terminus function intracellularly while losing extracellular function, truncated IFNs IFNα1(69–189)R9 and IFNβ(100–187)R9 with nine arginines (R9) for cell penetration were expressed in a bacterial system and purified. As controls, these truncations were also expressed without R9. Both IFNα1(69–189)R9 and IFNβ(100–187)R9 possessed antiviral activity against EMCV, while the same constructs without R9 for cell penetration lacked antiviral activity. R9 alone also lacked antiviral activity. This is consistent with previous studies that showed that intracellularly expressed IFNα possessed antiproliferative and antiviral activity (38). The truncation studies, however, are not subject to the argument that somehow the intracellular IFN may have leaked out of the cell and interacted with the extracellular receptor domains, since the truncations were not functional in terms of extracellular induced antiviral activity.

There are over 20 different isoforms of type I IFNs and they all function through the same heterodimeric receptor complex (21, 52, 53). In addition to their similar antiviral activities, these IFNs vary with respect to anticellular and cytotoxic (apoptotic) effects. In this regard, IFNβ is the treatment of choice for relapsing/remitting multiple sclerosis (MS) (14, 54). Further, it has been shown that higher doses of IFNβ result in better therapeutic efficacy (55), but undesirable toxic side-effects of flu-like symptoms, liver damage, and bone marrow suppression limit the dose (56). Differences in type I IFN toxicity (apoptosis) by different IFNs were shown to be due to differential extracellular IFN receptor recognition, where greater receptor occupancy due to higher binding affinity contributed to the toxic effects (13). This observation has been confirmed by others (57).

**Figure 3:**
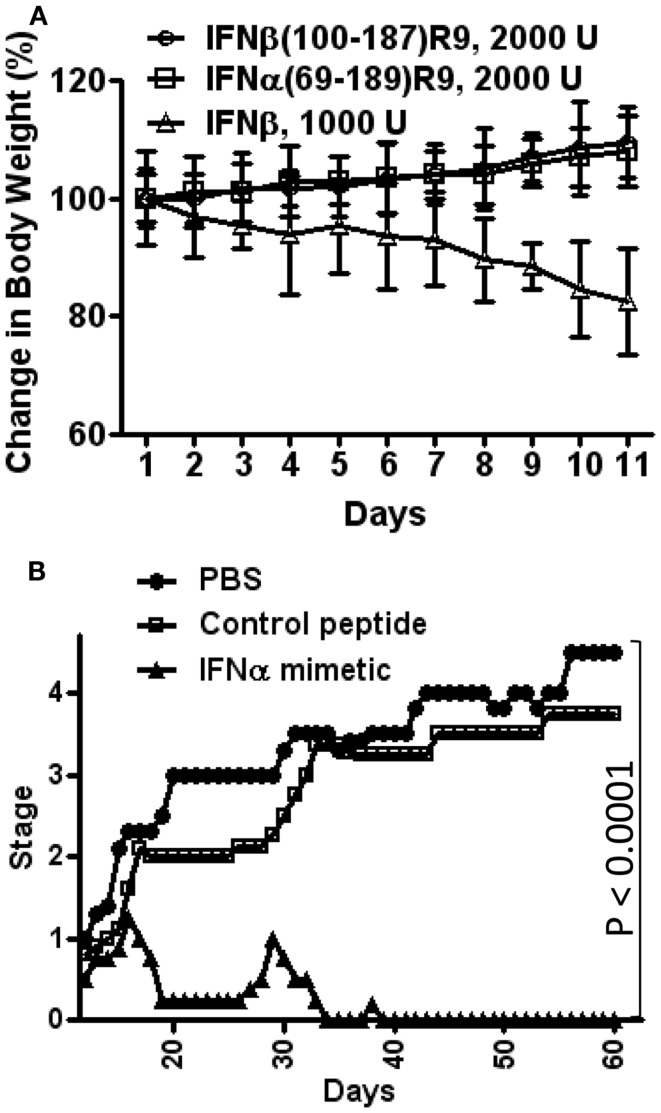
**Type I IFN mimetics protect against EAE, but lack the toxicity associated with the intact IFN**. **(A)** Weight loss comparison. Mice were injected i.p. with IFNβ (Δ, 1000 U/mouse); IFNβ(100–179)R9 (○), 2000 U (200 μg); or IFNα1(69–189)R9 (□), 2000 U (200 μg) on alternate days. Activity refers to antiviral units (U). Note that IFNβ injected mice had a greater than 15% loss of weight by day 11, while therapeutic mimetic injected mice gained weight. **(B)** N-terminal truncated type I IFN mimetic, IFNα1(69–189)R9, ameliorates EAE, a mouse model of MS. SJL/J mice (*n* = 5) were injected i.p. with PBS (●), IFNα1 mimetic IFNα1(69–189)R9 (▲, 15 μg/mouse), or mimetic absent cell-penetrating R9, IFNα1(69–189) (□, 15 μg/mouse) every other day starting from day 12 post-immunization of mice with bovine myelin basic protein. Mean severity of disease was graded as follows: 0, normal; 1, loss of tail tone; 2, hind leg weakness; 3, paraparesis; 4, paraplegia; 5, moribund; and 6, death. See Ahmed et al. (17) for details.

For toxicity studies, mice were injected intraperitoneally on alternate days with IFNβ, IFNβ(100–179)R9, or IFNα1(69–189)R9, all of the same antiviral activity (2,000 units) (17). Injection of mice with IFNβ resulted in approximately 15% weight loss by day 10, while mice injected with the IFN mimetics gained weight, which is expected under normal growth conditions (Figure 3A) (17). A similar pattern of bone marrow suppression occurred as reflected by peripheral lymphocyte count. IFNβ was also pro-apoptotic, while the mimetics did not induce apoptosis. Thus, under conditions of the same antiviral activity, IFNβ was toxic and the type I IFN mimetics lacked symptoms associated with toxicity such as weight loss, lymphopenia, and cellular toxicity.

IFNα1(69–189)R9 was tested for its ability to therapeutically treat SJL/J mice for experimental allergic encephalomyelitis (EAE), a mouse model of MS, without the toxicity associated with IFNβ treatment. EAE replicates the symptoms of MS in humans, and is induced by immunization of mice with various forms of myelin components (58). It is thought that T cells play a key role in the initiation and perpetuation of the inflammation that is associated with EAE, and that microglia and macrophages are attracted to the sites of inflammation by cytokines that are released by the T cells (58). The knowledge of the specific cell types that are involved in the inflammatory processes of EAE and MS has led to the focus on specific targets for drug development (59). The hallmark of treatment has until recently been IFNβ, but there are undesirable side-effects such as flu-like symptoms and injection site reactions (59). It is therefore important to develop new therapies that are tolerable to patients and that act in a manner similar to that of IFN.

Immunization of mice with bovine myelin basic protein where cellular infiltration into the CNS has occurred by day 12 was used to test the truncated IFNs (17). SJL/J mice were injected intraperitoneally with saline, IFNα1(69–189)R9, or the control peptide, IFNα1(69–189), 15 μg each every other day starting from day 12 post-immunization with MBP. The IFNα mimetic with the R9 reduced paralysis essentially completely, while the mice treated with saline or the mimetic lacking R9 developed paraplegia (Figure 3B) (17).

These mimetic results would suggest, as with binding studies mentioned above, that it is the IFN signal at the receptor extracellular domain that is responsible for their toxic effects, while antiviral and anti-EAE (MS) effects are associated with the intracellular actions that are retained by the IFN mimetics without the associated toxicity. It is important to emphasize that the IFN mimetics are products of the non-canonical model presented here.

## Endocytosis and Nuclear Transport of IFNγ and its Receptor: Learning from the EGF Receptor

Probably the most challenging conceptual aspect of the IFN signaling described here is the movement of a protein such as IFNGR1 from the plasma membrane to the nucleus. The mechanism of endocytosis of IFNGR1 and the relationship of this to the activation and nuclear translocation of STATα was examined to deal with this challenge (60). In untreated WISH cells, both receptor subunits IFNGR1 and IFNGR2 were constitutively localized within caveolae-like microdomains isolated from the plasma membrane. However, treatment of cells with IFNγ resulted in rapid migration of IFNGR1 but not IFNGR2 from these microdomains. Filipin treatment, which specifically inhibits endocytosis from caveolae-like microdomains, inhibited the nuclear translocation of IFNγ and IFNGR1 as well as the tyrosine phosphorylation and nuclear translocation of STAT1α, but did not affect the binding of IFNγ to WISH cells. In the Jurkat T lymphocyte cell line, which does not express caveolin-1, nuclear translocation of IFNGR1 and STAT1α were similarly inhibited by filipin pretreatment. Both IFNGR1 and IFNGR2 were associated with lipid microdomains in Jurkat cells, but only after stimulation with IFNγ, suggesting that IFNGR subunits are recruited to lipid microdomains by IFNγ binding in lymphocytes (Jurkat) in contrast to their constitutive presence in epithelial (WISH) cells. Treatment of cells where clathrin-dependent endocytosis is blocked did not inhibit either the activation or nuclear translocation of STAT1α nor the nuclear translocation of IFNγ and IFNGR1. Lipid microdomains were also independently shown by others to play a key role in IFNγ receptor endocytosis (61).

In another study of type I and type II IFN receptor endocytosis, both IFNα and IFNγ receptors were shown to be internalized by a classical clathrin-and dynamin-dependent endocytic pathway (62). Lipid-based microdomains were also shown to play a role in STAT activation and biological activity by IFNγ, but not by IFNα. Overall, it appears that lipid microdomains play a key role in IFNγ endocytosis and signaling, but this may not be the case for type I IFNs.

Epidermal growth factor receptor (EGFR) is a transmembrane glycoprotein that possesses intrinsic or receptor tyrosine kinase activity (63, 64). This differs significantly from IFNγ receptor, which has receptor-associated tyrosine kinase activity via JAK1 and JAK2. There are, however, similarities between the two receptors in that both EGFR and IFNGR1 undergo nuclear translocation following interaction with ligand. Both EGFR and IFNGR1 also function as transcription/co-transcription factors at promoters of genes that they activate and both have co-factors associated with them in the nucleus, analogous to steroid/steroid receptor (SR) signaling (11, 15, 65).

Considerable insight has been gained concerning the retrograde trafficking of EGFR from the cell membrane into the nucleus. Specifically, this system has been particularly useful in providing insight into how a plasma membrane protein with a hydrophobic transmembrane sequence migrates through the nuclear pore complex and functions as a transcription/co-transcription factor at promoters of activated genes. Upon treatment of MDA-MB-468 breast cancer cells with epidermal growth factor (EGF), confocal immunofluorescence revealed that EGFR underwent retrograde movement to the Golgi and the ER (endoplasmic reticulum) where the N-terminus was within the lumen of the Golgi/ER and the C-terminus was exposed to the cytoplasm (66). Retrograde trafficking was blocked by brefeldin A or dominant negative mutants of the small GTPase ARF (ADP-ribosylation factor), of which both treatments resulted in disassembly of the COPI (coat protein complex I) to the Golgi. It was concluded that the COPI regulated retrograde vesicular trafficking of EGFR from the Golgi to the ER. It was further shown that treatment of MDA-MB-468 cells with EGF resulted in trafficking of biotinylated cell-surface EGFR from the ER to the INM (inner nuclear membrane) through the nuclear pore complex, while maintaining its membrane-bound state (66, 67). It was confirmed that membrane-associated importin β regulated EGFR nuclear transport to the INM as well as to the nucleus/nucleoplasm. EGF was associated with EGFR through the retrograde transport pathway. Perhaps the most novel aspect of this study was the demonstration that Sec61β was found to be present in the INM and to associate with EGFR. Sec61β is a well-known ER-associated translocon that has previously been shown to be required for EGFR nuclear translocation (2). Translocons are conserved protein-conducting channels in eukaryotes. Knockdown of Sec61β expression reduced the level of EGFR in the nucleoplasm portion with concomitant accumulation in the INM (63, 67). Thus the Sec61β translocon played an unexpected critical role in the release of membrane-anchored EGFR from the lipid bilayer of the INM to the nucleus. These findings provide insight into the mechanism of nuclear transport of a membrane-bound full-length protein that functions as a transcription/co-transcription factor.

Fibroblast growth factor (FGF) and its receptor (FGFR) have previously been reviewed in detail concerning early studies of nuclear events (68–70). FGF and FGFR can provide further insight into nuclear transport mechanisms in terms of possible differences from EGFR in endocytic events. Briefly, FGFs are a family of approximately 20 different growth factors and/or isoforms. They interact at the cell-surface with FGF receptor tyrosine kinase, of which there are at least four different genes. Like EGF/EGFR, both FGF and FGFR undergo nuclear translocation in cells treated with FGF. It has been shown that FGF1 but not FGFR1 possessed a polycationic NLS, ^21^NYKKPKL (71). Endocytosis results in the exposure of the cytoplasmic domain of FGFR1 to the cytosol, while the N-terminus with associated FGF1 is in the endosomal lumen. It has been shown that FGF1 can penetrate the membrane of the endosomal vesicle to reach the cytoplasm (70). If the FGF1/FGFR1 complex undergoes nuclear translocation via the FGF1 NLS, the FGF1 would have to bind either to the receptor cytoplasmic domain or to another molecule that also binds FGFR1. For FGF1 translocation from the exogenous FGFR1 binding site to the cytosol and the nucleus, the C-terminal tail of the cytoplasmic domain of FGFR1 was required (72). This constituted approximately 50 amino acids downstream of the kinase domain of FGFR1. This finding is similar to that for the IFNγ binding site on the IFNGR1 cytoplasmic domain (42). Thus, it is tempting to interpret this as evidence of a cytoplasmic binding site on FGFR1 for FGF1.

Recent studies have provided more insight into how FGF1 and FGFR1 undergo nuclear translocation via a focus on FGF1. Leucine-rich repeat containing 59 (LRRC59) was identified as an intracellular binding partner of FGF1 (73). Following translocation of FGF1 across the endosomal membrane into the cytosol, it was shown to bind to LRRC59 on the cytosolic side of the ER membrane, which is crucial to FGF1 nuclear import. In this study, it was shown that the NLS of LRRC59 mediates the interaction with nuclear importins α and β, which are responsible for the import of proteins into the nucleus via binding to the NLS. It was proposed that this complex is transported along the continuous membranes of the ER, outer nuclear membrane (ONM), nuclear pore membrane, and the INM. Since FGFR1 is required for FGF1 nuclear import it would suggest that FGFR1 is part of this complex, but FGFR1 was not followed in this study. Other studies have suggested that importin β but not importin α is involved in the nuclear translocation of FGFR1 (64).

Consistent with the above studies, FGFR1 has been shown not to use the retrograde pathway of EGFR for nuclear import (64, 73). Membrane-associated importin β was involved in EGFR nuclear import, while cytosolic importins were required for FGFR. However, unlike the suggested scenario for FGF1 above, the comparison of FGFR1 to EGFR indicated that FGFR1 does not bind to the INM as determined by confocal microscopy (64). Thus, more details must be worked out with FGFR1 movement from the plasma membrane to the nucleus in a non-retrograde pathway that may or may not be linked to that of FGF1 nuclear import. Thus, testing for FGFR1 at the FGF1/LRRC59 complex would help provide more insight to FGFR1 transport to the nucleus in the context of FGF1 transport.

## Steroid Signaling: The Template for Non-Canonical IFNγ Signaling

In a search for a precedent, it seems that IFNγ, and probably EGF and FGF have similarities to that of SR signaling. We now provide an overview of steroid hormone (SH)/SR signaling to point out these similarities. SRs are a major subset of nuclear receptors. Basically, synthesis of SHs occurs in the adrenal cortex and in gonads (74). SHs are derivatives of cholesterol that are biosynthesized through various biochemical pathways. This involves the conversion of cholesterol into pregnenolone, which is subsequently converted into 17-hydroxypregnenolone and progesterone. 17-Hydroxypregnenolone gives rise to testosterone, which can be converted into estradiol via reduction. By a series of specific hydroxylations, progesterone gives rise to cortisol and aldosterone.

**Figure 4:**
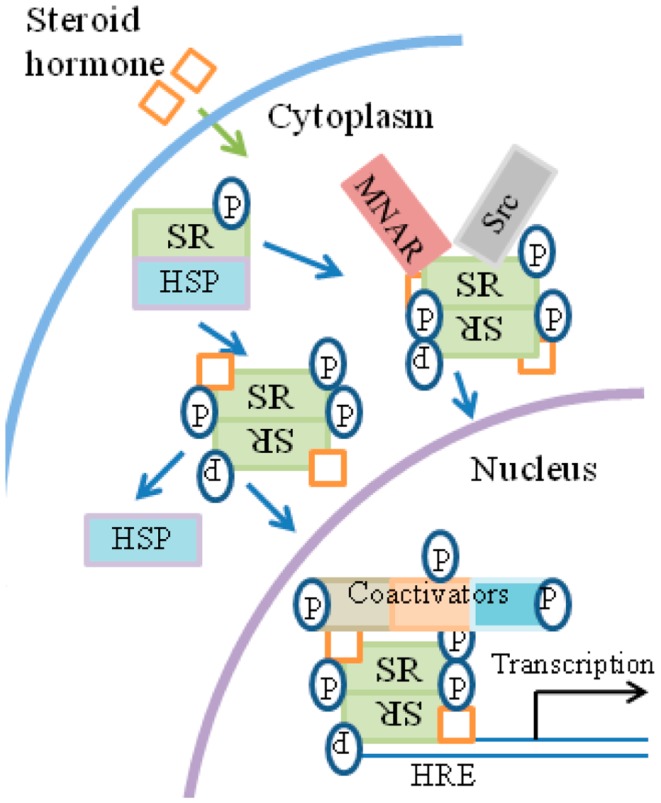
**General overview of steroid signaling**. (1) Ligand binds to the receptor in the cytosol. (2)The receptor functions as a transcription/co-transcription factor. (3) Co-activators such as kinases are associated with the ligand/receptor complex, which translocates to the nucleus. (4) The complex binds to response elements at specific genes. (5) Some of the co-factors of the complex, such as tyrosine and non-tyrosine kinases, are involved in the specific epigenetic events that accompany specific gene activation. HRE, hormone-response element; MNAR, modulator of non-genomic action of estrogen receptor; P, phosphotyrosine and non-phosphotyrosine kinase activity by co-factors and kinases.

Broadly, the current model of SH signaling is as follows and is summarized in Figure 4. SH binds to SRs located in the cytoplasm or nucleus of the cell. In the absence of hormone, SR monomers are associated with heat shock proteins (HSPs) and usually possess some basal level of phosphorylation. Upon binding of hormone, SRs dissociate from HSPs, dimerize and translocate to the nucleus where they bind to HREs (hormone-response elements) at genes that are activated by SHs. The complex of SH/SR recruits a series of co-activator complexes to both regulate target gene transcription as well as the associated epigenetic events that accompany such activation. Site-specific phosphorylation of receptors occurs subsequently to hormone binding with varied kinetics, depending on the kinase and the target in the receptor complex.

The kinases, although not the only components of the receptor-associated co-activator complexes, are important for their action on members of the complex, as well as for specific epigenetic events of gene activation and thus act on histones as well as on members of the receptor complex. Many of the SH phosphorylation sites contain serine/threonine/proline motifs involving proline-specific kinases, such as the cyclin-dependent kinases and MAPKs (75–77). Tyrosine kinases such as Src have also been shown to participate in SR signaling in the nucleus. SRs similarly cross-talk with receptor tyrosine kinases such as EGFR. EGFR family members are important targets in some of the most prevalent and difficult cancers, such as non-small cell lung carcinoma (78, 79).

In addition to their presence in the cytoplasm, a subset of SRs is also membrane-associated through an S-palmitoylation linkage to the inner side of the plasma membrane (76, 80). The membrane-associated SR may be in some cases the same as cytoplasmic SR, but this is not universally agreed upon. Membrane SR is involved in activation of MAPK and PI3K/Akt kinases.

In addition to the kinase-type activators described above, there are also so-called primary SRCs (SR co-activators), of which three are the most prominent (74). SRC proteins are recruited to hormone-bound SRs and bind through their LXXL motifs. SRCs recruit secondary co-activators, such as the histone acetyltransferase p300/CBP, the histone methyltransferases PRMT1 (protein arginine *N*-methyltransferase 1) and CARM1 (co-activator-associated arginine methyltransferase 1), and the chromatin remodeling complex SWI/SNF. These secondary co-activators modify the chromatin and bridge the SR complex with the general transcription machinery. Although the various kinases are present, just how they associate with SR and SRCs is not precisely known. One does come up with, however, the general picture of SH/SR-co-activator complexes where the co-activators may be grouped as primary as the case for the SRCs or secondary as the case for the histone transferases. If one were to restrict primary co-activators to the SRCs, then the kinases could also possibly be called secondary co-activators.

A comparison of IFNγ signaling in Figure 1B and SH signaling in Figure 4 suggests the following similar features. Ligand associates with the receptor intracellularly. In the case of IFNγ, first there is extracellular binding to IFNGR1 and then intracellular binding in conjunction with the endocytosis. SH penetrates the plasma membrane and binds the cytoplasmic SR. In both cases the receptors function as transcription/co-transcription factors. Co-activators are associated with the ligand/receptor complex. An overview of similarities between IFNGR1, IFNAR, EGFR, FGFR, and SR systems is presented in Table 1. Currently, much more is known concerning the SH/SR complex than the IFNγ/IFNGR1 or type I IFN/IFNAR complexes, but STATs and JAKs are associated in the cytoplasm and the nucleus. In both cases, the ligand-receptor-co-activator complex binds to response elements of genes that are specifically activated. Some of the co-factors, such as the kinases, are involved in specific epigenetic events for both systems. We do not feel that IFNs are a special case with respect to protein ligands with associated tyrosine kinase activity or with receptor tyrosine kinases, as EGFR and FGFR have similarities to the IFNs in receptor involvement in nuclear aspects of gene activation. We further feel that all of the cytokines, hormones, and growth factors that use the JAK/STAT pathway are likely to also share these similarities. In our view the template for all of this resides in the SH/SR system of specific gene activation.

**Table 1:** **Receptors as coordinators of complex formation and function in genetic and epigenetic changes in gene activation**.

Component	Signaling systems				
	IFNγ	IFNα	EGF	FGF	SH
Ligand	IFNγ	IFNα, β, ε, ω, k, or τ	EGF, TGFα	FGF	SH
	• Activates receptor	• Activates receptor	• Activate receptor	• Activates receptor	• Activates receptor
	• Provides NLS (40, 41)	• Provides NLS (17, 11)		• Provides NLS	
Receptor	IFNGR1	IFNAR1 IFNAR2	EGFR	FGFR1, FGFR2	SR
	• TF/Co-TF (15) IFNGR2	• Translocate to nucleus (17, 11)	• TF/Co-TF	• TF/Co-TF	• TF/Co-TF
	• Moves JAK2 to IFNGR1 (42)	• Provides NLS (81)	• Provides NLS	• TK activity for epigenetic modification	Platform for co-activators
			• TK and other kinase activity for epigenetic modification		
JAKs	• STAT activation	• STAT activation	• STAT activation	• STAT activation	
	• Epigenetic modification (16)	• Epigenetic modification (17)	• Epigenetic	• Epigenetic	
STATs	STAT1α	STATs 1 and 2	STATs 1, 3, 5	STAT5	STAT5
	• TF in activated state (16)	• TF in activated state (82)	• TFs	• TFs	• TF for Progesterone Receptor (84)
	• Heterochromatin stabilizer in non-phosphorylated state (36)				
Other associated nuclear kinases and co-factors	MAP kinase Erk1/2 F-κB (7)	MAP kinase Erk1/2 NF-κB (7)	MAP kinase Src (83)	MAP kinase Rsk1 (84)	MAP kinase SRC 1, 2, 3 (75), Msk1 and Erk (75)

*In IFNγ, EGF, FGF, and SR systems similarities in terms of steps involved in specific gene activation are indicated. Erk, Extracellular receptor kinase; Co-TF, Co-transcription factor; MAP Kinase, Mitogen activated protein kinase; Msk1, Mitogen, and stress activated kinase 1; Rsk1, Ribosomal S6 kinase 1; SH, steroid hormone; SR, steroid receptor; Src, cytoplasmic tyrosine kinase; SRC, steroid receptor co-activators; TF, transcription factor; TK, tyrosine kinase. Detailed references for the other players are provided in the text*.

## Conclusion

Our current understanding of cytokine signaling focuses in particular on the JAK/STAT pathway where activated STATs are responsible for specific gene activation even in the case of functionally different cytokines using the same STAT transcription factors. More recently, activated JAKs have been shown to perform important epigenetic functions, but such functions have not been coordinately coupled to the STAT transcription factors. It is possible that some of the observed differences are due to the use of different cell types and tissues by the studies referenced in this review. In the presence of this void, we have developed a non-canonical model of IFN signaling that takes the above events into account. This model bears some similarity to SH/SR signaling and has been useful in the development of IFN mimetics. It is our view that the model can be readily tested in the context of the various genetic and epigenetic aspects of cytokine signaling where linkage of genetic and epigenetic events is sought.

## Conflict of Interest Statement

The authors declare that the research was conducted in the absence of any commercial or financial relationships that could be construed as a potential conflict of interest.
